# JUN and PDGFRA as Crucial Candidate Genes for Childhood Autism Spectrum Disorder

**DOI:** 10.3389/fninf.2022.800079

**Published:** 2022-05-16

**Authors:** Heli Li, Xinyuan Wang, Cong Hu, Hao Li, Zhuoshuo Xu, Ping Lei, Xiaoping Luo, Yan Hao

**Affiliations:** ^1^Division of Child Healthcare, Department of Pediatrics, Tongji Hospital, Tongji Medical College, Huazhong University of Science and Technology, Wuhan, China; ^2^Tongji Medical College, Huazhong University of Science and Technology, Wuhan, China; ^3^Department of Immunology, School of Basic Medicine, Tongji Medical College, Huazhong University of Science and Technology, Wuhan, China; ^4^Department of Pediatrics, Tongji Hospital, Tongji Medical College, Huazhong University of Science and Technology, Wuhan, China

**Keywords:** autism, JUN, PDGFRA, cerebellum, DEGs

## Abstract

Autism spectrum disorder (ASD) is a complex neurodevelopmental disorder, characterized by marked genetic heterogeneity. In this study, two independent microarray datasets of cerebellum of ASD were integrative analyzed by NetworkAnalyst to screen candidate crucial genes. NetworkAnalyst identified two up-regulated genes, Jun proto-oncogene (JUN) and platelet derived growth factor receptor alpha (PDGFRA), as the most crucial genes in cerebellum of ASD patients. Based on KEGG pathway database, genes associated with JUN in the cerebellum highlight the pathways of Th17 cell differentiation and Th1 and Th2 cell differentiation. Genes associated with PDGFRA in the cerebellum were found enriched in pathways in EGFR tyrosine kinase inhibitor resistance and Rap1 signaling pathway. Analyzing all differentially expressed genes (DEGs) from the two datasets, Gene Set Enrichment Analysis (GSEA) brought out IL17 signaling pathway, which is related to the expression of JUN and PDGFRA. The ImmuCellAI found the elevated expression of JUN and PDGFRA correlating with increased Th17 and monocytes suggests JUN and PDGFRA may regulate Th17 cell activation and monocytes infiltrating. Mice model of maternal immune activation demonstrated that JUN and PDGFRA are up-regulated and related to the ASD-like behaviors that provide insights into the molecular mechanisms underlying the altered IL17 signaling pathway in ASD and may enable novel therapeutic strategies.

## Introduction

Autism spectrum disorder (ASD) is a multifactorial central nervous system (CNS) disorder that is characterized by impairments in social communication and repetitive and restrictive behaviors and interests (Lauritsen, 2013). Although the pathogenesis is complex, ASD is considered a highly heritable disorder with an estimated heritability of 50–90% (Xie et al., 2020). Genome-wide association studies (GWASs) have demonstrated various risk loci for ASD such as *FOXP1* at 3p13, *ATP2B2* at 3p25.3 [Autism Spectrum Disorders Working Group of the Psychiatric Genomics Consortium, 2017(Autism Spectrum Disorders Working Group of The Psychiatric Genomics Consortium., 2017)]. However, the risk genes of ASD remain largely unknown because only a small part of phenotypic variation can be explained by GWAS-identified loci. Therefore, gathering additional evidence using other methods, such as gene expression, is vital.

The mechanisms underlying the association between ASD candidate genes and the neurobiology of autism are complex because genes encode multiple highly complex functions in different regions of the brain. To date, numerous studies have demonstrated associations between brain abnormalities and ASD (Maynard et al., 2021; Vacher et al., 2021; Rubin, 2022). Furthermore, studies have increasingly highlighted the cerebellum as a pathological area of the brain in ASD (Carta et al., 2019; Sathyanesan et al., 2019; Kelly et al., 2020; Vacher et al., 2021). Moreover, the co-occurrence of early behavioral defects in most neurodevelopmental disorders, which span motor, sensory, cognitive, and emotional domains, indicates that abnormalities in the cerebellum are the main determinant of ASD (Sathyanesan et al., 2019). Evidence that directly supports this idea comes from the clinical characteristics of non-motor deficits in areas of executive function, memory, and language in children with cerebellar malformations (Tavano et al., 2007) or those who have undergone cerebellar tumor resection (Levisohn et al., 2000).

Among the factors associated with CNS disorders, it is curious that non-CNS disruptions such as allergies (Akdis, 2021), gastrointestinal disorders (Bresnahan et al., 2015), and mitochondrial dysfunctions (Giulivi et al., 2010) are also associated with autism. Specifically, prenatal exposure to maternal immune activation (MIA) is considered a key environmental risk factor for ASD (Han et al., 2021; Mirabella et al., 2021; Xu et al., 2021). Animal models for ASD conducted by prenatal exposure, including prenatal infection, valproic acid, propionic acid, poly (I:C) or lipopolysaccharides exposure, can cause ASD-like behaviors, such as specific impairments in social interaction, communication and repetitive behaviors (Ergaz et al., 2016; Mintál et al., 2022). The MIA mice model is one of the most frequently studied models of ASD in which a single prenatal exposure to poly (I:C) results in behavioral, neurological, and immunological abnormalities of the offspring (Knuesel et al., 2014). Recent studies have reported the involvement of T helper 17 (Th17) lymphocyte activity and its core mechanism of inflammation, IL-17 signaling pathway in ASD processing (Nadeem et al., 2020; Ellul et al., 2021; Willyard, 2021). Activation of IL-17 signaling pathway and elevated interleukin-17A (IL-17A) levels have been implicated in studies of ASD patients (Reed et al., 2020). Moreover, in MIA mice offspring, increased populations of Th17 cells and elevated IL-17A levels have been observed (Kim et al., 2017). In addition, BTBR *T*^+^*Itpr*3^tf^/J (BTBR) mice have also been used as an ASD animal model due to their low levels of sociability and high levels of repetitive grooming (Ryan et al., 2019), which have obviously Th17 related immune responses (Bakheet et al., 2017; Nadeem et al., 2018). Activated Th17 cells in the ASD brain activate the immune response and recruit further immune cells, such as monocytes and T cells, by elevating IL-17 secretion (Sie et al., 2014; Hoogenraad and Riol-Blanco, 2020). Therefore, Th17 cells may play a vital role in the development of ASD. However, the role of Th17 imbalance in the cerebellum of ASD has not been fully elucidated.

We applied an integrated bioinformatics analysis to explore key regulators using NetworkAnalyst, a web-based visual analytics platform for comprehensive profiling, meta-analysis and systems-level interpretation of gene expression data (Zhou et al., 2019). The utility of NetworkAnalyst for identifying differentially expressed genes (DEGs) and pathways has recently been demonstrated (Santiago and Potashkin, 2015; Li et al., 2020; Dai et al., 2022). For example, network analyses have identified HNF4A and PTBP1 as effective biomarkers for Parkinson's disease (Santiago and Potashkin, 2015) and ELAVL1 and APP as crucial candidate genes for Crohn's disease (Li et al., 2020).

Here, we conducted a network-based bioinformatics analysis to screen DEGs in the cerebellum of ASD patients, which was followed by Kyoto Encyclopedia of Genes and Genomes (KEGG) pathway enrichment analysis (https://www.genome.jp/kegg) (Kanehisa and Goto, 2000), gene set enrichment analysis (GSEA), and interaction network analysis of DEGs by NetworkAnalyst. We identified JUN and PDGFRA, which have previously been implicated in regulating T cell differentiation (Schraml et al., 2009; Piccaluga et al., 2014). Furthermore, GSEA data showed that IL-17 signaling-associated gene signatures were enriched, and ImmuCellAI suggested that Th17 cells were increased in the cerebellum, which could aid our understanding of the role of JUN and PDGFRA in ASD.

## Materials and Methods

### Microarray Source

Gene expression data from microarray studies of ASD were downloaded from the Gene Expression Omnibus (GEO) (https://www.ncbi.nlm.nih.gov/geo/) (Edgar et al., 2002) by using the terms “Autism,” “ASD” and “cerebellum.” All microarray studies analyzed in this study are listed in Table 1. Only two microarray studies using RNA prepared from human cerebellum were selected for subsequent integrative analysis from 24 datasets. GSE28521 from GPL6883 included 10 ASD patients and 11 typical development controls (TD) (Voineagu et al., 2011). The cerebellums (vermis) for all ASD and TD of GSE28521 were obtained from Autism Tissue Program (ATP, www.brainbank.org/) and the Harvard Brain Bank (http://www.autismtissueprogram.org). The GSE38322 from GPL10558 included 8 ASD patients and 8 TD (Ginsberg et al., 2012). The cerebellums (cerebellar hemispheric cortex) for all ASD and TD of GSE38322 were obtained from ATP, the Harvard Brain Bank and the National Institute for Child Health and Human Development (NICHD) Brain and Tissue Bank (www.btbank.org). All datasets were pre-processed using the log2 transformation and quantile normalization by the *r* package.

**Table 1:** Gene expression datasets used in this study.

**Disease**	**Datasets**	**Platform**	**Organism**	**Brain tissue**	**Cases**	**Controls**	**References**
**Exploration**							
ASD	GSE28521	GPL6883	Homo sapiens	Cerebellum	10	11	Voineagu et al., 2011
ASD	GSE38322	GPL10558	Homo sapiens	Cerebellum	8	8	Ginsberg et al., 2012
Validation							
ASD	GSE28521	GPL6883	Homo sapiens	Frontal cortex	16	16	Voineagu et al., 2011
ASD	GSE28521	GPL6883	Homo sapiens	Temporal cortex	13	13	Voineagu et al., 2011
MIA mice	GSE34058	GPL6247	Rattus norvegicus	Brain	9	10	Oskvig et al., 2012
MIA mice	GSE117327	GPL23038	Mus musculus	Medial prefrontal cortex	5	8	www.ncbi.nlm.nih.gov/geo/
BTBR T+tf/J	GSE62594	GPL13912	Mus musculus	Cerebellum	8	8	Shpyleva et al., 2014

The separate analyses using GEO2R (Smyth, 2004) of the two datasets was performed according to the commonly used threshold parameters (|logFC| > 1.0 and adjusted *p* < 0.05), accessible *via* the National Center for Biotechnology Information website (https://www.ncbi.nlm.nih.gov/).

### Integrated Network-Based Bioinformatics Analysis

NetworkAnalyst is a web server with the function of creating tissue specific (including cerebellum) PPI networks, gene regulatory networks, gene co-expression networks (Zhou et al., 2019). We performed integrated bioinformatics analysis by NetworkAnalyst, in accordance with the protocol of how to perform and visualize meta-analysis on multiple gene expression data (Xia et al., 2015).

The network-based bioinformatics analysis used the Fisher's method with a significance level of *p* < 0.05 to combine *p* values from the multiple datasets by NetworkAnalyst according to the pipeline described previously (Santiago and Potashkin, 2015). Fisher's method is a widely used statistical approach to combine *p* values from different studies (Tseng et al., 2012). The cerebellum-specific protein-protein interaction of the DEGs was constructed, and the KEGG pathway enrichment was analyzed using a hypergeometric test by NetworkAnalyst, which have human tissue-specific PPI data from the DifferentialNet database (Xia et al., 2015).

### Gene Set Enrichment Analysis

Pre-ranked GSEA was performed using fgsea (Fast Gene Set Enrichment Analysis), a NetworkAnalyst module powered by the *r* package (Zhou et al., 2019). All DEGs ranked by fold changes and results were visualized by interactive heatmaps.

### Abundance of Infiltrating Immune Cells in the Cerebellum Samples

ImmuCellAI (http://bioinfo.life.hust.edu.cn/ImmuCellAI#!/) can estimate the abundance and differences in the infiltration of 24 types of immune cells (Miao et al., 2020), which has been well used for cancer and non-cancer tissue such as brain (Li et al., 2022). We analyzed the immune cell types, including 18 T-cell subtypes and 6 other immune cells: B cell, NK cell, Monocyte cell, Macrophage cell, Neutrophil cell and DC cell, in all cerebellum samples. The immune scores were predicted by calculating the ssGSEA enrichment score of expression deviation profile per cell type of each dataset using ImmuCellAI.

### The Human Protein Atlas (HPA)

HPA (https://proteinatlas.org/) includes various protein levels of human gene expression profile information (Uhlén et al., 2015). In this study, we examined the expression of JUN and PDGFRA in the brain regions of the GTEx human brain RNA-Seq dataset and the HPA mouse brain RNA-Seq dataset from HPA.

### Mice

C57BL/6 mice were purchased from HFK Bioscience (Beijing, China) and housed under specific pathogen-free conditions (Morton et al., 2008). Timed pregnancies in mice (6–8 weeks) were implemented by housing a male and female pair overnight, and pregnant mice received intraperitoneal (IP) injections of 20 mg/kg Poly I:C (#P9582, Sigma) at embryonic day 12.5 (E12.5). All male offspring from each litter were used for behavior tested at 6 weeks of age, with 3–5 litters used for each treatment group. Female mice were mated with male mice overnight and checked daily for pregnancy.

### Open Field Test

The open field experiment is a method to evaluate the depression-like behavior of experimental animals in a novel environment. Mice were placed in an open field (43.2 × 43.2 cm). Activity and position of each mouse within the open field was measured for 5 mins. Data were analyzed by the MED Associates' Activity Monitor Data Analysis software.

### Three-Chamber Test

Social behavior was measured using the three-chambered arena (40 × 60 cm). In Phase 1, the test mouse could explore the whole arena (habituation). In Phase 2, an empty cage was placed in one of the side chambers, and one unfamiliar mouse (stranger 1) was placed into a cage in the other side chamber. The test mouse was gently placed in the central chamber, and allowed to explore for 10 min. In Phase 3, the stranger 1 was replaced with a different unfamiliar mouse (stranger 2) and the stranger 1 was placed in the other cage. Time spent in each chamber and the sniffing zones was recorded by Ethovision XT 10 system (Noldus), a most widely applied video tracking software that tracks and analyzes the behavior, movement, and activity of any animal (Grieco et al., 2021).

### RNA Extraction and Quantitative Real-Time PCR (qRT-PCR)

After behavior tested, total RNA from entire cerebellum of MIA male offspring (6 weeks, *n* = 6) was extracted using the TriZol Reagent (Invitrogen), and cDNA was synthesized using the RevertAid First Strand cDNA Synthesis Kit (Thermo Fisher Scientific). qRT-PCR were achieved using a SYBR green real-time PCR kit (Toyobo, Osaka, Japan); reactions were run on a LightCycler (Bio-Rad Laboratories, Hercules, CA, USA). mRNA levels were normalized to GAPDH, and fold changes were determined using the 2–Δ Δ Ct method.

The primers are used as follows: 5′-GCGGACCTTATGGCTACAGT-3′ and 5′-CCCGTTGCTGGACTGGATTA-3′ (JUN); 5′-TCGCCAAAGTGGAAGAGACC-3′ and 5′-TCACCAACAGCACCAACACT-3′ (PDGFRA); 5′-TCATCCCTCAAAGCTCAGCG-3′ and 5′-TGCGCCAAGGGAGTTAAAGA-3′ (IL-17A); 5′-TCTCCACACCTATGGTGCAA-3′ and 5′- CAAGAAACAGGGGAGCTGAG-3′ (GAPDH).

### Cytokine's Analysis

The concentration of IL-17A in supernatants of lysed cerebellar samples was determined by the CBA Mouse Th1/Th2/Th17 Cytokine Kit from Becton Dickinson following the manufacturer's instructions.

### Statistical Analysis

All basic statistical analyses, which included the Mann-Whitney test, Pearson correlation, and Spearman correlation and rank correlation were calculated using the *r* software, a free software environment for statistical computing and graphics (Li et al., 2020). A *p*-value < 0.05 and *r* > 0.3 or *r* < −0.3 was considered statistically significant and relevant. Data were presented as means and standard deviations (SDs) or medians and quantiles depending on the distribution of data.

## Manuscript

### Seven DEGs Were Selected Using Integrated Bioinformatics Analysis

**Figure 1:**
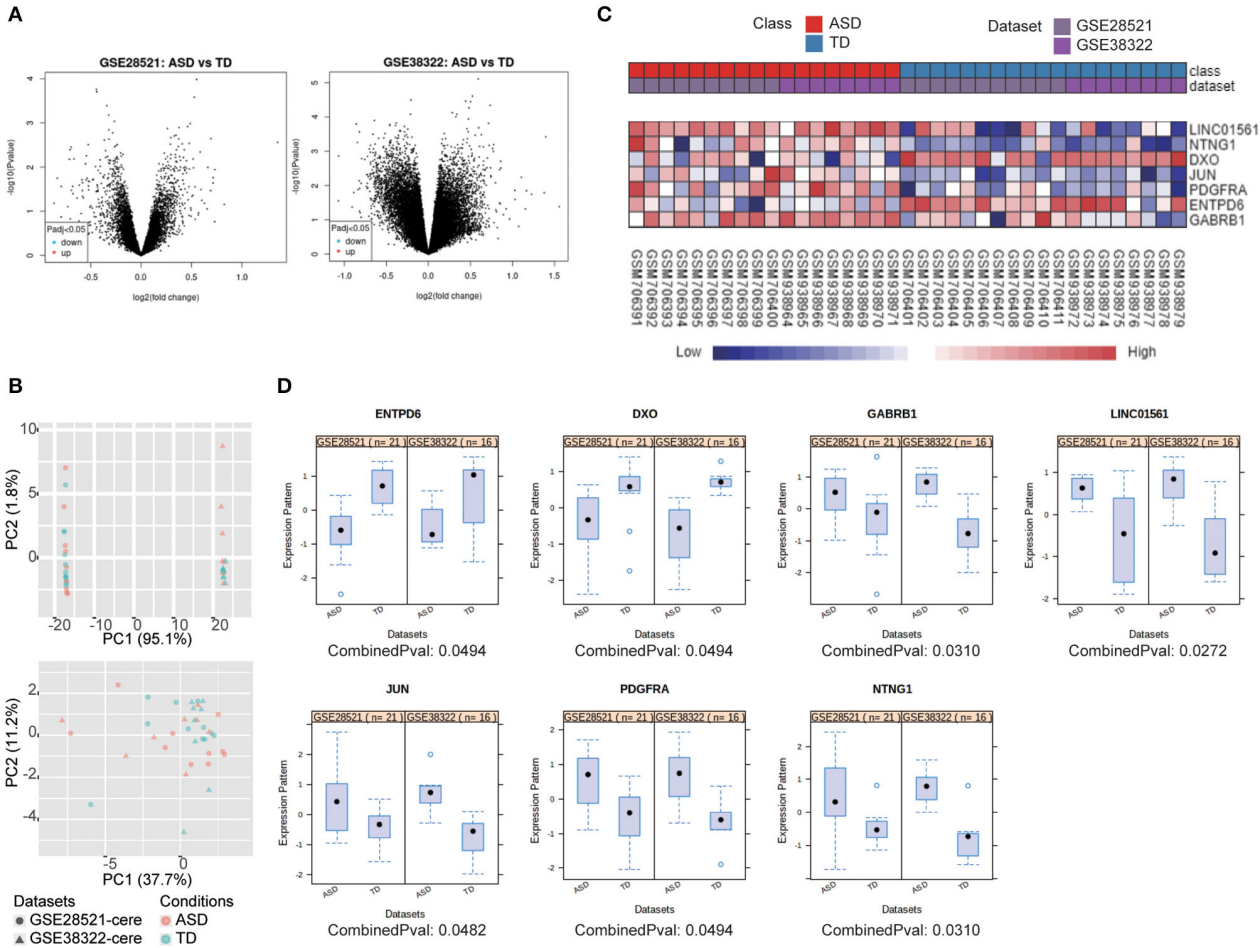
7 DEGs were selected using integrated bioinformatics analysis. **(A)** Volcano plot of DEGs between ASD patients and TD for GSE28521 (left) and GSE38322 (right) were generated by GEO2R (adjusted *p* < 0.05). **(B)** PCA plots all datasets without (upper) and with batch effect adjustments (lower). **(C)** Heat map representation of the DEGs across the two microarrays identified from the integrated analysis. **(D)** The expression of DEGs in ASD and TD from the two datasets represented by scatterplots.

Two microarray studies (Table 1) were analyzed using NetworkAnalyst to screen DEGs in the cerebellum of ASD patients. The separate analyses using GEO2R of the two datasets revealed that no DEGs could be identified in either dataset (Figure 1A). The datasets were then processed to eliminate the batch effect, and parametric empirical Bayes frameworks provided by the ComBat function were performed using NetworkAnalyst. The PCA plots with and without batch effect adjustments indicated that the processed data were more comparable (Figure 1B).

Integrated bioinformatics analysis identified gamma-aminobutyric acid type A receptor subunit beta1 (GABRB1), long intergenic non-protein coding RNA 1561 (LINC01561), JUN, PDGFRA, and netrin G1 (NTNG1) were upregulated, and decapping exoribonuclease (DXO) and ectonucleoside triphosphate diphosphohydrolase 6 (ENTPD6) were downregulated in ASD compared with typical development controls (TD). Heat map visualization of these seven genes of the two datasets is provided in Figure 1C, and individual boxes for each gene expressed in ASD and TD are shown in Figure 1D.

### JUN and PDGFRA Are Hub Genes Identified Using Network-Based Analysis

**Figure 2:**
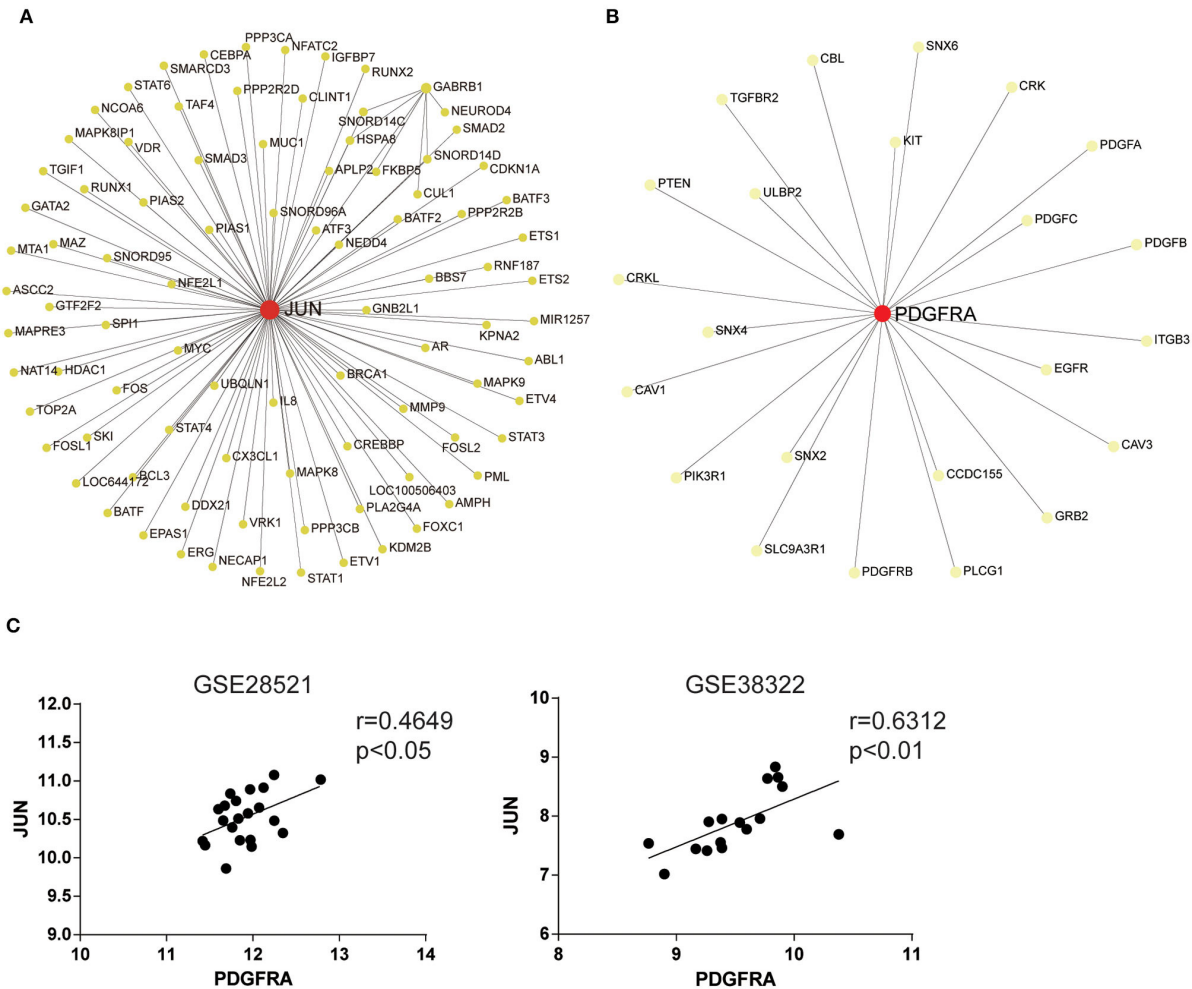
JUN and PDGFRA are hub genes identified using network-based analysis. Network analysis of all DEGs in the cerebellum of ASD patients. The first **(A)** and the second **(B)** network of genes regulated in the cerebellum of ASD patients. The colors of nodes are positively correlated with the number of node-neighbors. **(C)** Scatterplots with regression lines. Correlation between JUN and PDGFRA expression in the cerebellum of ASD patients and TD.

To explore abnormal biological processes in the cerebellum of patients with ASD, we entered all DEGs into a network-based analysis using NetworkAnalyst. Degree of centrality (DC) and betweenness (BC) showed that the seven identified DEGs exist as two obvious networks in the cerebellum. DC is the number of connections of a node with other nodes. BC measures number of shortest paths going through the node (Han et al., 2004). Nodes with higher degree or betweenness values are potentially important hubs in the network. The most highly ranked node in the first network was JUN (DC = 85; BC = 3,978.5; Figure 2A), followed by GABRB1 (DC = 7; BC = 351.5). The results of the KEGG pathway enrichment analysis of the network (including 91 genes) are shown in Table 2, which include Th17 cell differentiation and Th1 and Th2 cell differentiation.

**Table 2:** KEGG analysis of the genes involved in the first network.

**Term**	**Count**	**Gene**	***P* value**
Pathways in cancer	25/530	4.7%	1.08e-12
Hepatitis B	15/163	4.7%	3.93e-11
Th17 cell differentiation	13/107	12.1%	3.93e-11
HTLV-I infection	16/219	7.3%	1.19e-10
Th1 and Th2 cell differentiation	10/92	10.9%	4.96e-08

The most highly ranked node in the second network was PDGFRA (DC = 23; BC = 253; Figure 2B), followed by SNX2 (DC = 1; BC = 0). The results of the KEGG pathway enrichment analysis of the network (including 24 genes) are shown in Table 3, which include focal adhesion, EGFR tyrosine kinase inhibitor resistance, and the Rap1 signaling pathway. Moreover, the expression of JUN was strongly positively associated with PDGFRA in the two datasets (Figure 2C). These results suggest that JUN and PDGFRA both play important roles in the cerebellum of ASD patients and indicate the presence of several signaling pathways in which JUN and PDGFRA work as the most important hub gene.

**Table 3:** KEGG analysis of the genes involved in the second network.

**Term**	**Count**	**Gene**	***P* value**
Focal adhesion	14/199	7.0%	3.65e-6
EGFR tyrosine kinase inhibitor resistance	10/79	12.7%	1.77e-5
Rap1 signaling pathway	12/206	5.8%	2.85e-5
Glioma	9/75	12.0%	3.1e-5
Prostate cancer	9/97	9.3%	6.01e-5

### IL-17 Signaling Pathway Is Enriched in the Cerebellum of ASD Patients

**Figure 3:**
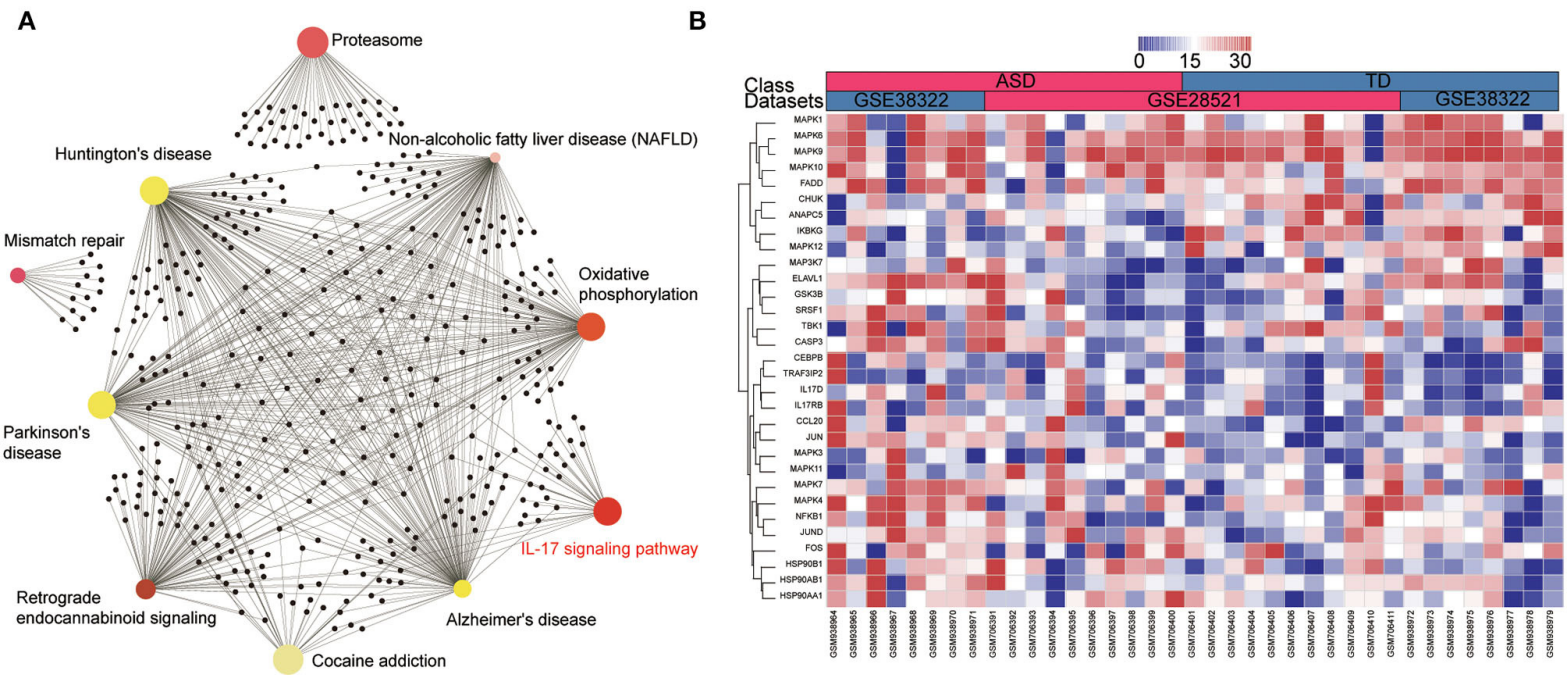
IL-17 signaling pathway is enriched in the cerebellum of ASD patients according to the GSEA analysis. **(A)** The enrichment network of DEGs identified using network-based analysis, the colors of nodes are positively correlated with the fold change of DEGs in the signaling pathway. **(B)** GSEA-generated heatmaps of genes in the IL-17 signaling pathway in the two datasets.

To further explore the mechanisms of ASD, pre-ranked GSEA was employed to analyze the two microarray datasets by selecting fold change as the gene ranking method. As shown in Figure 3A, the GSEA analysis suggested that the IL-17 signaling pathway was the only enriched pathway in the top 10 gene sets across the two datasets. The GSEA-generated heatmaps showed that the core enrichment genes in the IL-17 signaling pathway were upregulated in the ASD patients in these datasets (Figure 3B). Results revealed that the IL-17 signaling pathway may act as a crucial role in the development of ASD.

### Upregulation of JUN and PDGFRA Correlated With Genes in the IL-17 Signaling Pathway

**Figure 4:**
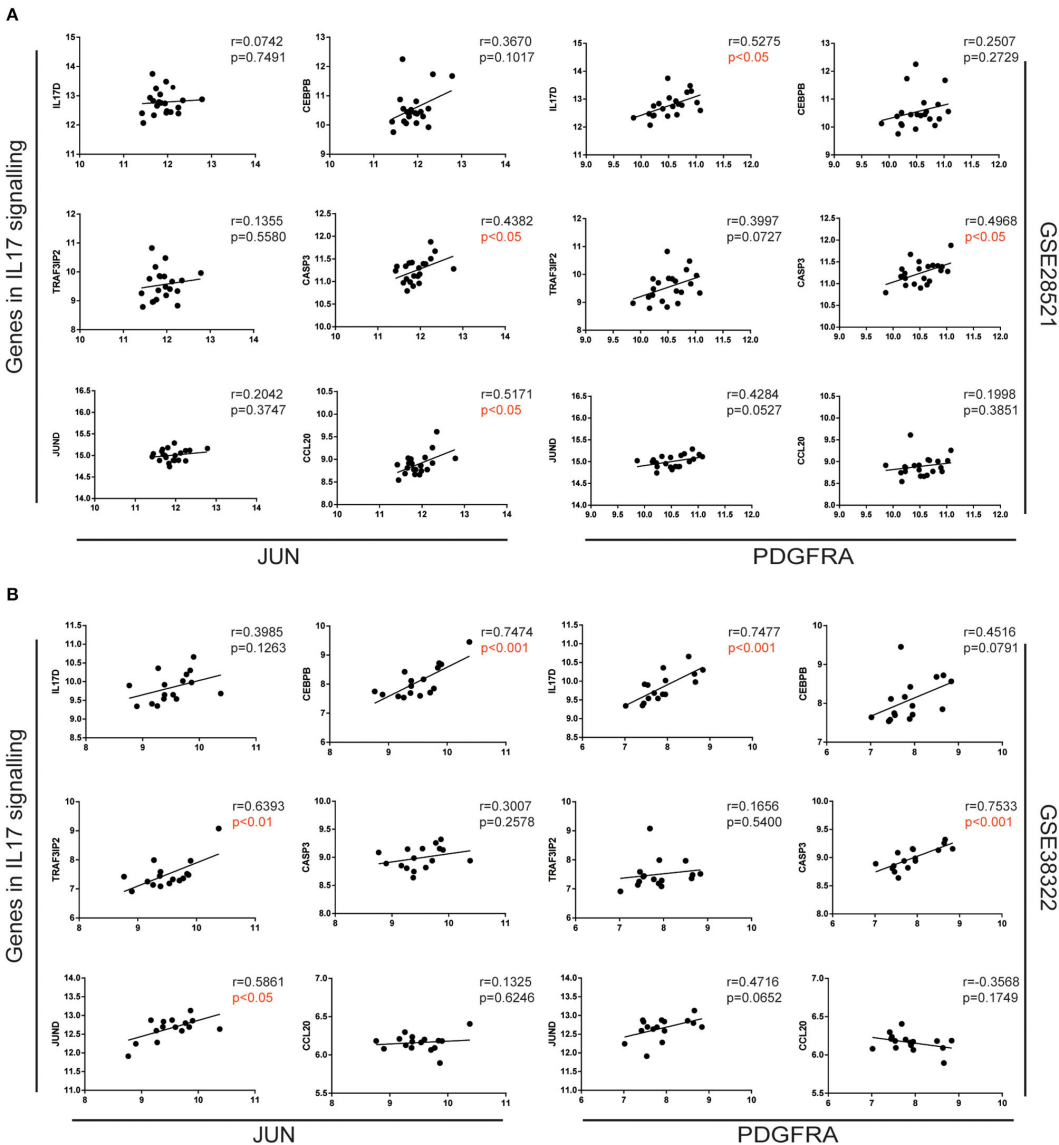
Correlation of JUN and PDGFRA correlated with genes in the IL-17 signaling pathway. Correlation between the gene expression of JUN (left) and PDGFRA (right) and selected genes in the IL-17 signaling pathway, analyzed using expression data from GSE28521 **(A)** and GSE38322 **(B)** datasets are illustrated by scatterplots with regression lines.

To further explore the association between JUN, PDGFRA, and the IL-17 signaling pathway in ASD patients, we analyzed the correlation between JUN and PDGFRA and the genes in the IL-17 signaling pathway (Figure 3B) using Pearson correlation. Various genes in the IL-17 signaling pathway, including IL-17D, CEBPB, TRAF3IP2, CASP3, JUND, and CCL20, were associated with the upregulation of JUN and PDGFRA (Figures 4A,B), suggesting a direct relationship between JUN, PDGFRA, and IL-17 signaling in the cerebellum of ASD patients.

### Upregulation of JUN and PDGFRA Correlates With the Abundance of Th17 and Monocyte Subsets in the Cerebellum of ASD Patients

**Figure 5:**
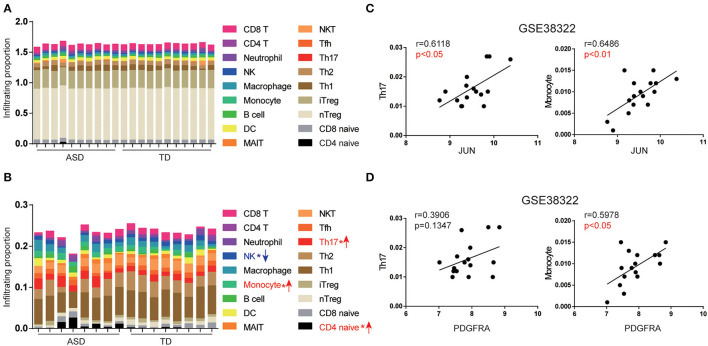
Expression of JUN and PDGFRA correlated with Th17 and monocyte subsets in the cerebellum of ASD patients. The abundance of immune cells by group and the whole cohort of ASD patients in GSE28521 **(A)** and GSE38322 **(B)** (*p < 0.05). The association between JUN **(C)**, PDGFRA **(D)**, and infiltration scores of Th17 and monocytes of the cerebellum were analyzed using the expression data from GSE38322. Scatterplots were shown with regression lines.

The infiltration scores of immune cells were evaluated using ImmuCellAI, based on the gene expression of each sample. We analyzed the proportions of the 18 types of immune cells in the cerebellum of ASD patients and TD from GSE28521 (Figure 5A) and GSE38322 (Figure 5B). We found that Th17, CD4 naïve cells, and monocytes were more abundant in ASD patients than in TD in the cerebellum in GSE38322 (Figure 5B). However, the cerebellum samples from GSE28521 differed slightly between ASD patients and TD (Figure 5A). The inconsistency of the two datasets may be due to the slightly different regions of cerebellum of the two datasets. We further investigated the relationship between the expression of JUN and PDGFRA and the infiltration scores of Th17 and monocytes in the cerebellum from the GSE38322 dataset using Spearman correlation. As shown in Figures 5C,D, JUN expression was positively correlated with the infiltration scores of Th17 (*r* = 0.6118, *p* < 0.05) and monocytes (*r* = 0.6486, *p* < 0.01). Similarly, PDGFRA expression was positively correlated with the infiltration scores of Th17 (*r* = 0.3906, *p* = 0.1347) and monocytes (*r* = 0.5978, *p* < 0.05).

Th17 cells are activated and recruited by the brain of ASD patients to activate the immune response (Choi et al., 2016), which can recruit further immune cells, such as monocytes and T cells, by elevating IL-17 secretion (Sie et al., 2014). These results imply that upregulation of the expression of JUN and PDGFRA is associated with Th17 cell activation, which is likely through the IL-17 signaling pathway.

### Expression of JUN and PDGFRA Relates to Neurodevelopmental Disorders and Human ASD Risk Genes

**Figure 6:**
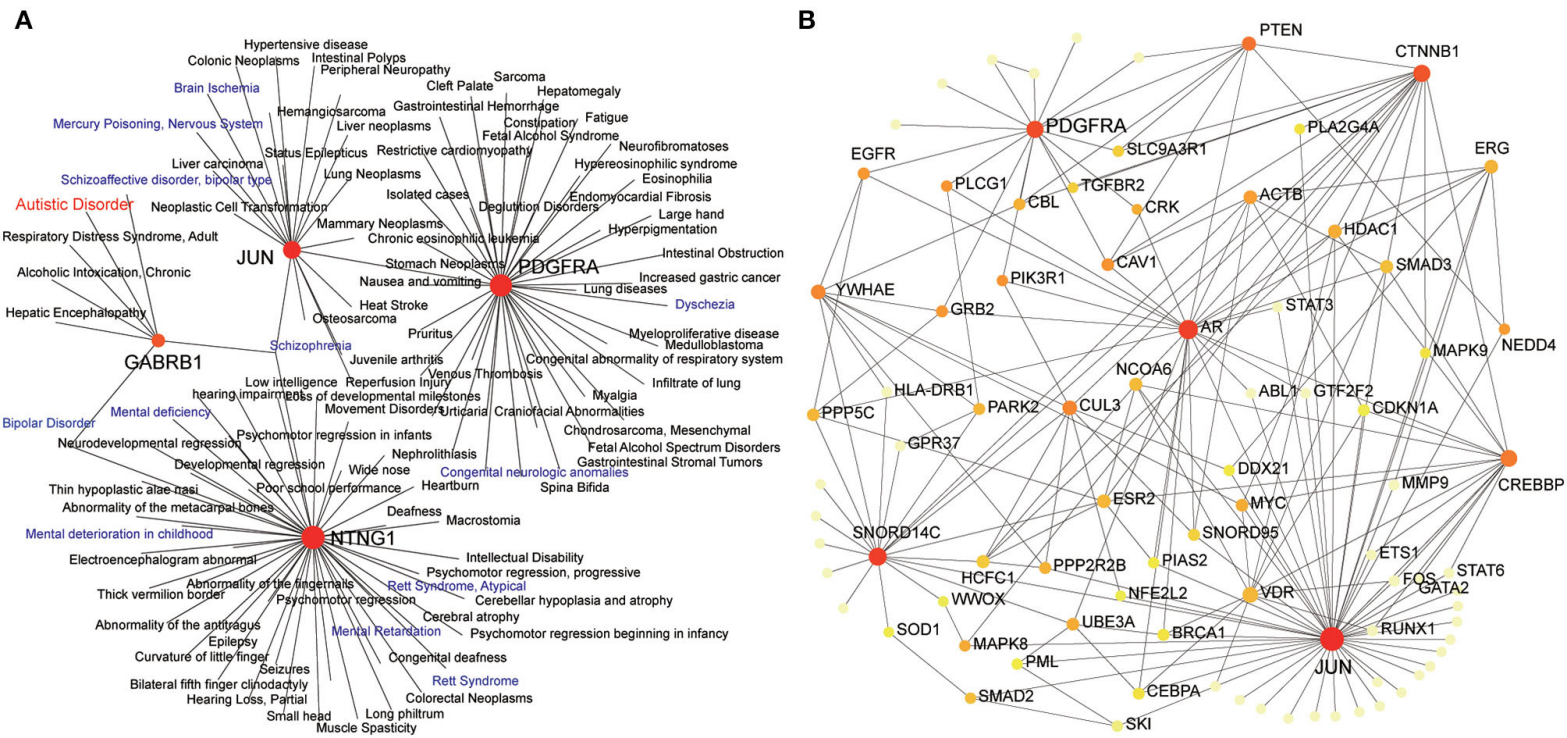
Expression of JUN and PDGFRA relates to neurodevelopmental disorders and human ASD risk genes. Zero-order interaction networks of JUN and PDGFAR with human diseases **(A)** and ASD risk genes from SFARI **(B)**. The colors of nodes are positively correlated with the number of node-neighbors.

To further explore the possible mechanisms of JUN and PDGFRA in ASD, gene–disease associations were analyzed by NetworkAnalyst. As shown in Figures 6A,B, JUN (DC = 19; BC = 3965), PDGFRA (DC = 40; BC = 3588), NTNG1 (DC = 47; BC = 4035) and GABRB1 (DC = 7; BC = 575) were associated with multiple neurodevelopmental disorders, including schizophrenia, bipolar disorder, Rett syndrome, seizures, and autistic disorder. We then analyzed the association of JUN and PDGFRA with human ASD risk genes from the Simons Foundation Autism Research Initiative (SFARI) database (Banerjee-Basu and Packer, 2010) by NetworkAnalyst. JUN (DC = 11; BC = 132.83) and PDGFRA (DC = 7; BC = 106.16) were both related to various verified ASD risk genes such as PTEN, CTNNB1 and EGFR.

### Evaluation of JUN and PDGFRA mRNAs in the Brain of ASD Patients

**Figure 7:**
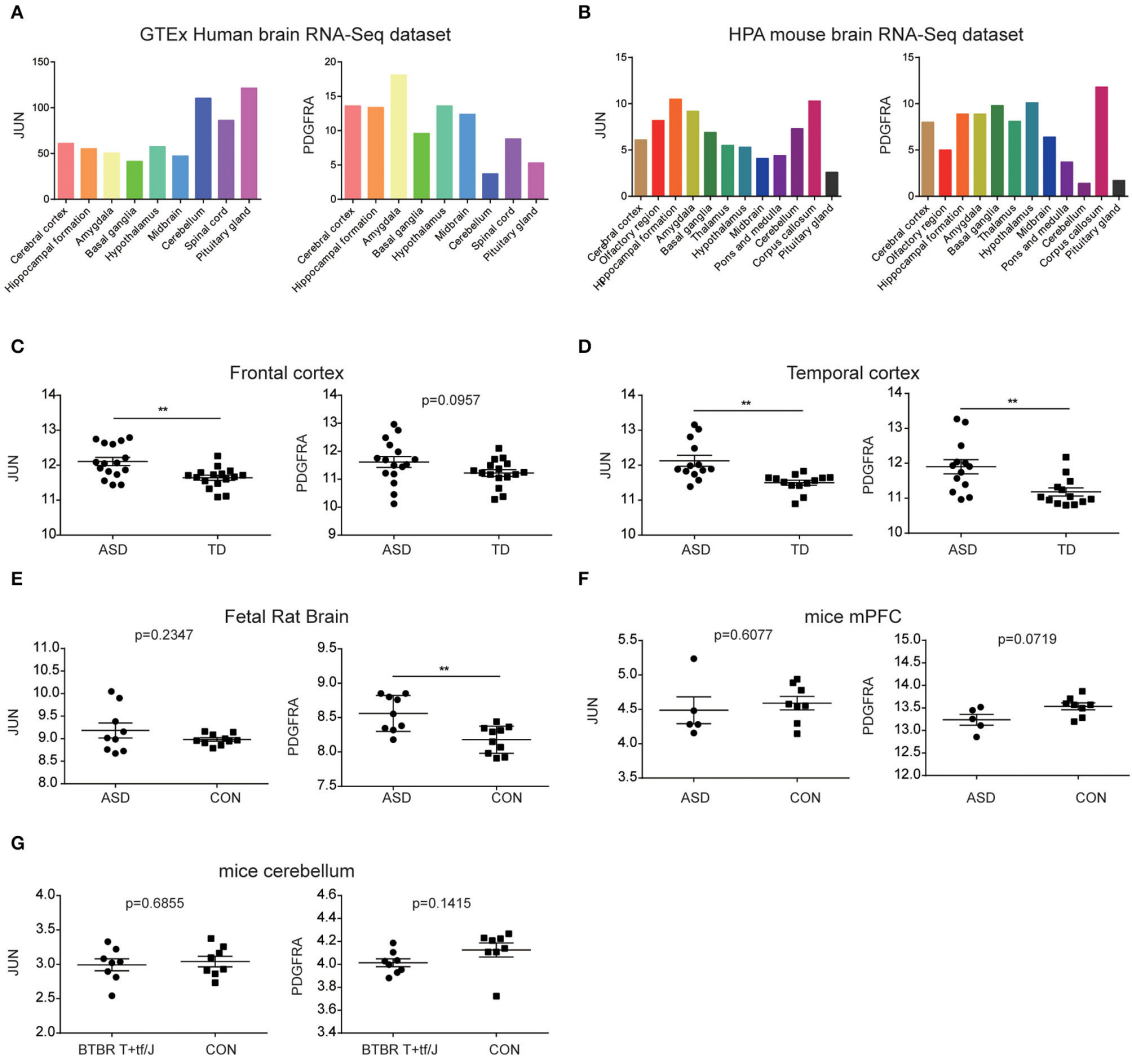
Evaluation of JUN and PDGFRA mRNAs in ASD brains. The expression of JUN and PDGFRA in the brain regions of the GTEx human brain RNA-Seq dataset **(A)** and the HPA mouse brain RNA-Seq dataset **(B)**. Expression of JUN and PDGFRA in the frontal **(C)** and temporal cortices **(D)** of samples from GSE28521. Expression of JUN and PDGFRA in the fetal rat brain from GSE34058 **(E)** and mPFC from GSE117327 **(F)**. **(G)** Expression of JUN and PDGFRA in the cerebellum of BTBR T+tf/J and control mice (CON) from GSE62594. **p < 0.01.

According to the information from the data of The Human Protein Atlas, JUN and PDGFRA can be detected in all brain's regions of humans and mice, and both have low region specificity (Figures 7A,B). Thus, we explored whether the expression of JUN and PDGFRA was altered in other regions of the brain in ASD. mRNA expression of JUN and PDGFRA was analyzed in the frontal and temporal cortices of ASD patients and TD from samples obtained from GSE28521. As shown in Figures 7C,D, JUN expression was significantly higher in the frontal and temporal cortices of ASD than that of TD, whereas PDGFRA was only upregulated in the temporal cortex of ASD patients. These results suggested the expression of JUN and PDGFRA may vary depending on brain region. We further explored the expression of JUN and PDGFRA in two relevant animal models of autism, the MIA and BTBR T+tf/J mouse models, which have been demonstrated to have increased Th17 cells and IL-17. PDGFRA, but not JUN, was upregulated in the fetal brains of MIA offspring (Figure 7E). Moreover, the expression of JUN and PDGFRA in the medial prefrontal cortex (mPFC) of MIA offspring and in the cerebellum of BTBR T+tf/J mice were similar to those of the control mice (CON) (Figures 7F,G). These results indicate that the expression levels of JUN and PDGFRA differed between brain regions and were primarily upregulated in the cerebellum of MIA offspring.

### Elevated Expression of JUN and PDGFRA in MIA Model ASD Mice

**Figure 8:**
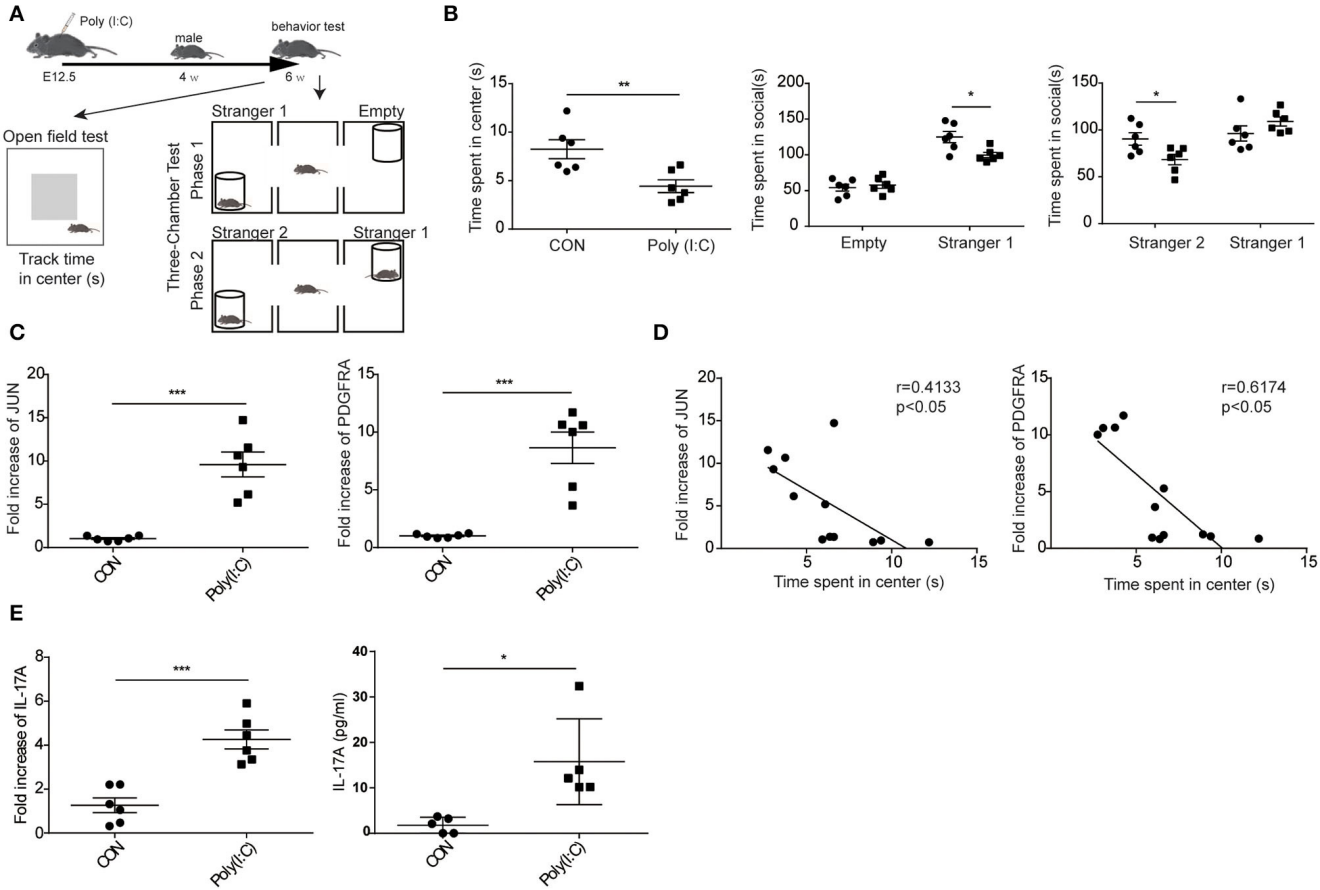
Elevated expression of JUN and PDGFRA in MIA model ASD mice. **(A)** Schematic representation of the procedures followed to establish the MIA model and the behavior experiment. **(B)** In the open field and social behavior tests, the poly (I:C) group showed significantly less time in the center area and socially interacting. Data are presented as means ± standard error of the mean (*n* = 6, *p < 0.05, **p < 0.01, ***p < 0.001). **(C)** qPCR for JUN, PDGFRA, and IL-17 signaling-associated gene, IL-17A, in the entire cerebellum of MIA male offspring (*n* = 6, *p < 0.05, **p < 0.01). Representative data from three independent experiments were shown. **(D)** In the open field tests, the association between the mRNA expression of JUN (right), PDGFRA (left), and the time spend in center of mice were analyzed (*n* = 12). Scatterplots were shown with regression lines. **(E)** The concentration of IL-17A in supernatants of lysed cerebellar samples examined by CBA kit.

To validate the hypothesis that the expression of JUN and PDGFRA is significantly increased in the cerebellum of ASD mice from MIA model. As shown in Figure 8A, the cerebellums were removed from the control and Poly(I:C) offspring at 6 weeks after behavior tested. Poly(I:C) offspring showed greater depression-like behavior and fewer social interactions with stranger 1 and stranger 2 than control offspring (Figure 8B). qPCR assays revealed that JUN, PDGFRA, and IL-17A mRNAs were significantly higher in the cerebellum of Poly(I:C) offspring compared with control (CON) (Figures 8C,E). Moreover, the time spend in center of mice were negatively correlated with the mRNA expression of PDGFRA (*r* = 0.6174, *p* < 0.05) and JUN (*r* = 4133, *p* < 0.05). These results suggested that the increased expression of JUN and PDGFRA was related to ASD development, which is likely *via* the facilitation of the IL-17 signaling pathway.

## Discussion

The pathogenesis of ASD is complex, and identifying the crucial regulators in the CNS is essential for understanding and improving the clinical management of ASD. Integrated analysis provides an unbiased method to identify and prioritize biologically central biomarkers for neurodegenerative diseases (Santiago and Potashkin, 2014). Here, we identified crucial candidate genes in the cerebellum of ASD patients using a network-based analysis of GSE28521 and GSE38322 and selected seven DEGs among these datasets. JUN and PDGFRA were identified as the most crucial genes by NetworkAnalyst. In addition, differential expression of the two hub genes was validated by other datasets and an ASD mouse model, which indicated that JUN and PDGFRA play a vital role in the development of ASD.

Network-based analysis identified JUN as the most crucial hub gene. JUN encodes c-Jun, also known as activator protein 1 (AP-1), which is a transcription factor that is considered to be the main regulator of neuronal death and regeneration (Coffey et al., 2002). The activity of c-Jun is regulated by phosphorylation, which is mediated by the c-Jun N-terminal kinase (JNK) family, including JNK1, JNK2, and JNK3 (Petrov et al., 2016). In the CNS, the activation of c-Jun is essential for regeneration after antiretroviral-induced peripheral neuropathy, whereas the activation of JNK3 causes neuropathic pain (Sanna et al., 2020). The activation of c-Jun caused by stressful stimuli is mediated by JNK2 and JNK3, which leads to cell death of cerebellar neurons (Coffey et al., 2002). Moreover, JNK3 is related to various neurodegenerative diseases, whereby the activation of JNK3 has impact on triggering apoptosis (Lin et al., 2017) and neuronal death in several neurodegenerative disorders (Antoniou et al., 2011). Recent research has indicated that JNK activation is heavily involved in the pathophysiological mechanism of ASD, based on its function in regulating basal dendrite development in cortical neurons (de Anda et al., 2012) and cognitive impairment (Pavlowsky et al., 2010). Additionally, JUN is also associated with the function of glutathione, which regulating proinflammatory cytokines, thus providing a significant impact on neuroinflammation (Bjørklund et al., 2020). Consistently, our gene-disease analysis indicated that JUN is associated with mercury poisoning and brain ischemia (Figure 6A), which are involved in neuronal death and result in the development of ASD (Nance et al., 2015; Kern et al., 2016). Although the exact mechanism is not clearly understood, these lines of evidence suggest a link between JUN and ASD.

PDGFRA, a classical proto-oncogene that encodes receptor tyrosine kinases that respond to platelet-derived growth factor (PDGF), was recently identified to impact the formation of the neural crest (Guérit et al., 2021). Notably, mice lacking PDGFRA cannot survive and present multiple defects in CNS (Andrae et al., 2008). Activated PDGFRA transduces the signals involved in multiple downstream pathways, including the PI3K/Akt, MAP kinase, and EGFR pathways (Snuderl et al., 2011; Paugh et al., 2013), which have all been implicated in the brain of ASD patients (Zhang et al., 2019; Hörnberg et al., 2020; Mossa et al., 2021). However, studies on PDGFRA in ASD are limited. Numerous researchers have found that both adult and pediatric forms of glioblastoma (GBM) are related to the activation of PDGFRA (Guérit et al., 2021). Furthermore, EGFR kinase, the most prominent oncogenic target for GBM, has been disappointing after inhibited by drugs in clinical trials (Kondapalli et al., 2015). Notably, we found the pathway enrichment of genes in the second PDGFRA network as the core EGFR tyrosine kinase inhibitor resistance in the cerebellum of ASD patients (Table 3). There is an increasing recognition that GBM and ASD share the same fundamental pathophysiological mechanisms at the cellular and molecular levels (Prasad and Rao, 2015). Therefore, our work on exploring the hub genes in ASD may function as a novel mediator for both ASD and GBM, which could facilitate the development of treatment strategies for ASD as well as brain cancers.

The results from the GSEA highlight the importance of the IL-17 signaling pathway in the cerebellum of ASD patients (Figure 3A). IL-17 is the core member of the IL-17 family of cytokines, which includes IL-17A, IL-17B, IL-17C, IL-17D, IL-17E, and IL-17F (Kawaguchi et al., 2004). IL-17, also known as IL-17A, is the signature cytokine produced by Th17 cells and is the only cytokine that is prominently upregulated in MIA offspring (Hoogenraad and Riol-Blanco, 2020). Recent studies have shown that IL-17 is a key contributor to neurodevelopmental abnormalities in MIA offspring (Choi et al., 2016). Interestingly, the pathway enrichment of genes from the first network in the cerebellum of ASD patients with JUN as the core also highlighted the Th17, Th1, and Th2 cell differentiation pathways (Table 2), which play vital roles in the pathogenesis of ASD (Ahmad et al., 2017; Bakheet et al., 2017). Notably, the ImmuCellAI data indicated that Th17 and monocyte proportions were significantly increased in the cerebellum, which may have been caused by the function of IL-17 in immune response activation and immune cell recruitment (Sie et al., 2014). It is reasonable to presume that the IL-17 signaling pathway may function as a vital regulator of ASD pathology in the cerebellum. In addition, our work also demonstrated the close connection of JUN and PDGFRA with genes in the IL-17 signaling pathway (Figure 4). Moreover, although the mechanisms of PDGFRA in the mediation of the IL-17 signaling pathway remain unclear, our results indicate that EGFR, which is closely related to PDGFRA in the cerebellum of ASD (Figure 2B), plays an essential role in the activation of Th17 (Hardbower et al., 2016). Thus, it will be worth exploring the link between JUN and PDGFRA and the IL-17 signaling pathway and investigating their roles in the progression of ASD.

It is well-known that there are significant differences in the structure and function of various brain regions. Because of the low region specificity of JUN and PDGFRA in the brain (Figures 7A,B), we further evaluated their mRNA in other brain regions. The relative abundance of JUN mRNA was upregulated in the frontal and temporal cortices of ASD patients (Figures 8C,D). However, the association between the relative abundance of JUN and PDGFRA did not reach statistical significance in BTBR T+tf/J mice. One possible explanation is that the mechanism of Th17 activation in the cerebellum may differ from that in BTBR T+tf/J and MIA mice. These results indicate that the upregulation of JUN and PDGFRA mRNA in the cerebellum are specific biomarkers for MIA offspring. In our animal model, we found an abundance of JUN and PDGFRA in the cerebellum, and the increase in IL-17A further supported the role of genes in ASD (Figure 8). However, the current study could not demonstrate the regulatory functions of JUN and PDGFRA on the IL-17 signaling pathway, and further investigation on the regulator of the IL-17 pathway and their connection to abnormal behaviors in an animal model is needed.

## Conclusion

Our study suggests that JUN and PDGFRA are crucial candidate genes in the cerebellum of children with ASD and highlights the roles of the IL-17 signaling pathway in the activation of the immune response in ASD. JUN may modulate the activation of Th17 cells in the cerebellum of ASD, whereas PDGFRA may be a key regulator that is associated with the EGFR pathway and Th17 activation. Our analysis provides valuable insight and further understanding of the mechanism of JUN and PDGFRA in the pathogenesis of ASD.

## Data Availability Statement

The original contributions presented in the study are included in the article/supplementary material, further inquiries can be directed to the corresponding author.

## Ethics Statement

The animal study was reviewed and approved by Hubei Provincial Animal Care and Use Committee.

## Author Contributions

HL, YH, and XL designed the study, analyzed the data, and wrote the manuscript. XW, CH, HL, ZX, and PL analyzed the data. YH provided funding. All authors contributed to the article and approved the submitted version.

## Funding

This work was supported by Huazhong University of Science and Technology Emergency Technology Research Project Response to COVID-19 (Grant Number 2020kfyXGYJ020) and Key Project of Independent Innovation Research Fund of Huazhong University of Science and Technology (Grant Number 2017KFYXJJ100).

## Conflict of Interest

The authors declare that the research was conducted in the absence of any commercial or financial relationships that could be construed as a potential conflict of interest.

## Publisher's Note

All claims expressed in this article are solely those of the authors and do not necessarily represent those of their affiliated organizations, or those of the publisher, the editors and the reviewers. Any product that may be evaluated in this article, or claim that may be made by its manufacturer, is not guaranteed or endorsed by the publisher.
